# Clinical and Molecular Features of Renal and Pheochromocytoma/Paraganglioma Tumor Association Syndrome (RAPTAS): Case Series and Literature Review

**DOI:** 10.1210/jc.2017-00562

**Published:** 2017-07-28

**Authors:** Ruth T. Casey, Anne Y. Warren, Jose Ezequiel Martin, Benjamin G. Challis, Eleanor Rattenberry, James Whitworth, Katrina A. Andrews, Thomas Roberts, Graeme R. Clark, Hannah West, Philip S. Smith, France M. Docquier, Fay Rodger, Vicki Murray, Helen L. Simpson, Yvonne Wallis, Olivier Giger, Maxine Tran, Susan Tomkins, Grant D. Stewart, Soo-Mi Park, Emma R. Woodward, Eamonn R. Maher

**Affiliations:** 1 Department of Medical Genetics, University of Cambridge and National Institute for Health Research Cambridge Biomedical Research Centre and Cancer Research UK Cambridge Centre, Cambridge CB2 OQQ, United Kingdom; 2 Department of Endocrinology, Cambridge University National Health Service (NHS) Foundation Trust, Cambridge CB2 OQQ, United Kingdom; 3 Department of Histopathology, Cambridge University NHS Foundation Trust and Cancer Research UK Cambridge Centre, Cambridge CB2 OQQ, United Kingdom; 4 West Midland Regional Genetics Laboratory, Birmingham Women’s NHS Foundation Trust, Birmingham B15 2TG, United Kingdom; 5 Manchester Centre for Genomic Medicine, St Mary's Hospital, Central Manchester University Hospitals NHS Foundation Trust, Manchester Academic Health Science Centre, Manchester M13 9WL, United Kingdom; 6 Haematology Oncology Diagnostic Service, Cambridge University NHS Foundation Trust, Cambridge CB2 OQQ, United Kingdom; 7 Division of Surgery and Interventional Science, University College London, Royal Free Hospital, London NW1 2BU, United Kingdom; 8 Department of Clinical Genetics, University Hospitals Bristol NHS Foundation Trust, Bristol BS2 8HW, United Kingdom; 9 Academic Urology Group, University of Cambridge and Cancer Research UK Cambridge Centre, Addenbrooke’s Hospital, Cambridge CB2 OQQ, United Kingdom

## Abstract

**Context:**

The co-occurrence of pheochromocytoma (PC) and renal tumors was linked to the inherited familial cancer syndrome von Hippel-Lindau (VHL) disease more than six decades ago. Subsequently, other shared genetic causes of predisposition to renal tumors and to PC, paraganglioma (PGL), or head and neck paraganglioma (HNPGL) have been described, but case series of non–VHL-related cases of renal tumor and pheochromocytoma/paraganglioma tumor association syndrome (RAPTAS) are rare.

**Objective:**

To determine the clinical and molecular features of non-VHL RAPTAS by literature review and characterization of a case series.

**Design:**

A review of the literature was performed and a retrospective study of referrals for investigation of genetic causes of RAPTAS.

**Results:**

Literature review revealed evidence of an association, in addition to VHL disease, between germline mutations in *SDHB, SDHC, SDHD, TMEM127*, and *MAX* genes and RAPTAS [defined here as the co-occurrence of tumors from both classes (PC/PGL/HNPGL and renal tumors) in the same individual or in first-degree relatives]. In both the literature review and our case series of 22 probands with non-VHL RAPTAS, *SDHB* mutations were the most frequent cause of non-VHL RAPTAS. A genetic cause was identified in 36.3% (8/22) of kindreds.

**Conclusion:**

Renal tumors and PC/PGL/HNPGL tumors share common molecular features and their co-occurrence in an individual or family should prompt genetic investigations. We report a case of *MAX*-associated renal cell carcinoma and confirm the role of *TMEM127* mutations with renal cell carcinoma predisposition.

## Teaser

We studied clinical and molecular features of renal tumor and pheochromocytoma tumor association syndrome by literature review and characterization of a large novel case series.

Causes for the occurrence of different tumor types in the same individual or in close relatives may include shared environmental exposures and /or inherited neoplasia disorders. Combinations of specific tumor types may strongly implicate specific inherited cancer syndromes (1). Thus the combination of pheochromocytoma (PC) and renal cell carcinoma (RCC) was recognized as a “form fruste” of von Hippel-Lindau (VHL) disease more than 60 years ago (2). RCC is the most common form of adult renal cancer, and ∼3% occurs from a hereditary disorder (3). PC and paraganglioma (PGL) are functional neuroendocrine tumors arising from the adrenal medulla (PC) or sympathetic ganglia (PGL) with an annual incidence of 2 to 8 per 1 million persons (4). The proportion of PC/PGL cases attributable to a genetic cause is at least 10-fold higher than for RCC (5); some genetic causes of PC/PGL also predispose to head and neck paraganglioma (HNPGL). Nevertheless, the combination of RCC and PC/PGL in a single individual or close relatives is rare and, if cases of VHL disease are excluded, clinical and molecular studies are limited mostly to anecdotal case reports (6–9).

In this study, we have investigated the genetic architecture of the clinical association (in the same individual or family) of a renal tumor and a PC/PGL/HNPGL without evidence of VHL disease [referred to here as non-VHL renal and pheochromocytoma/paraganglioma tumor association syndrome (RAPTAS)]. We undertook a comprehensive literature review and a retrospective study of a large case series of 22 probands (index cases) and 11 affected first-degree relatives (FDRs) referred to tertiary genetic services.

## Methods

### Case series

Details of patients referred for molecular genetic testing because of a suspected hereditary cause of PC/PGL or RCC over a period of 15 years (2001 through 2016) were reviewed and those with clinical (*e.g.*, in addition to PC/RCC, the presence of retinal or central nervous system hemangioblastoma, multiple renal or pancreatic cysts, pancreatic neuroendocrine tumors, endolymphatic sac tumors) or molecular evidence of VHL disease were excluded. Patients included had either (1) a personal history of PC/PGL/HNPGL and a renal tumor or (2) the presence of PC/PGL/HNPGL and RCC in FDRs (*e.g.*, PC in a proband and RCC in a parent). Patients meeting these criteria were classified as having non-VHL RAPTAS. Referral data from three UK National Health Service molecular diagnostic laboratories undertaking genetic testing were collated on a standardized pro forma and included sex, age at presentation, method of presentation (sporadic vs familial), location of tumor, presence of bilateral/multifocal disease, and evidence of malignancy. Molecular genetic testing information was also collected. Patients gave written informed consent to a research ethics committee–approved research study and/or data were collected as part of a molecular genetics service evaluation study.

### Molecular genetic testing of patients in case series

Some cases referred before 2011 had individual gene testing (*e.g.*, *VHL*, *SDHB*) but more recent cases were tested for a panel of up to 10 susceptibility genes (*SDHA*, *SDHB*, *SDHC*, *SDHD*, SDHAF2, *VHL*, *MAX*, *TMEM127*, *RET*, *FH*), mostly using a next-generation sequencing (NGS)-based assay described previously (10). All participants gave informed consent for clinical diagnostic genetic testing. NGS was performed using the Illumina or Ion Torrent platforms. On average, coverage depth of >20-fold was achieved for 98% of the regions sequenced. All pathogenic variants were confirmed by Sanger sequencing. Copy number changes in *VHL, SDHB, SDHC*, and *SDHD* were sought by multiple ligation probe analysis. Targeted tumor sequencing was performed on DNA extracted from four macro-dissected formalin-fixed paraffin-embedded tumor samples with a custom panel based on the Ion AmpliSeq™ Cancer Hotspot Panel v2 with additional bespoke content (Supplemental Table 4). The Covaris Adaptive Focused Acoustics™–based DNA extraction and purification from formalin-fixed paraffin-embedded tissue protocol was used and 20 ng of extracted DNA was sequenced. Library preparation was performed using an adapted Ampliseq on MiSEquation 2 primer protocol. Sequencing was performed on the Illumina MiSeq system.

### Bioinformatics and histology review

See the Supplemental data for more information.

### Literature review

A full review of the published literature on the genes reported to predispose to PC/PGL or RCC up to December 2016 was performed. This search was performed and included publications indexed in PubMed (http://www.ncbi.nlm.nih.gov/pubmed) up to June 2017. Search terms included *NF1, RET, MAX, EGLN1, EGLN2, MSH2, KIFIB, SDHAF2,MEN1*, *BAP1, CDC73, CDKN2B, FLCN, MET, PBRM1, PTEN, TSC1, TSC2, FH, SDHA*, *SDHB, SDHC, SDHD*, TMEM127, and *VHL* genes, hereditary, renal cell carcinoma, oncocytoma, kidney cancer, pheochromocytoma, and paraganglioma. In addition, the Human Gene Mutation Database (www.hgmd.cf.ac.uk) and the Leiden Open Variation Database (http://www.lovd.nl/3.0/home) were reviewed. The search results were interrogated to identify genetic causes of RAPTAS.

### MTS

We applied the previously described multiple primary tumor score (MTS) (11) to group A.

### Statistical analysis

Statistical tests were performed using SPSS. Summary statistics include mean and standard deviation (SD) for continuous variables and frequency and percentage for categorical variables. A two-sample *t* test was applied to parametric means and a Mann-Whitney test was applied as the nonparametric equivalent test. Fisher’s exact test was used to calculate the statistical difference between proportions of wild-type *versus* alternate allele reads.

## Results

### Case series demographics

Thirty-three individuals (16 males, 17 females) with PC/PGL/HNPGL and/or a renal tumor from 22 kindreds without clinical or molecular evidence of VHL disease met our criteria for the diagnosis of non-VHL RAPTAS. This cohort was subdivided into two groups: multiple tumor patients with a combination of PC/PGL/HNPGL + RCC (n = 12 probands; group A) and familial non-VHL RAPTAS cases with RCC or PC/PGL/HNPGL and an FDR with the alternative tumor type (n = 21 patients, 10 probands; group B).

#### Clinical features of group A: multiple tumor non-VHL RAPTAS cases

Twelve patients with a diagnosis of PC/PGL and a renal tumor were identified. The clinical details are summarized in Table 1. Seven cases had synchronous tumors and five metachronous. Mean age at diagnosis of first tumor was 55.3 years (SD, 19.4; range, 10 to 76 years). Four of five metachronous cases presented with PC/PGL/HNPGL and one patient was initially diagnosed with RCC. In most cases, a unilateral PC was present (75%, 9/12 patients), but there were two cases (16.6%) with HNPGL and one with an abdominal PGL. Most renal tumors were RCC (91.7%, 11/12 patients), but a renal oncocytoma was present in a patient without a germline mutation. One group A patient had been diagnosed with breast carcinoma, but no additional tumors such as gastrointestinal stromal, thyroid, or pituitary tumors were identified in group A or group B patients (Tables 1 and 2).

**Table 1.** **Clinical Features and Genetic Features of RAPTAS Patients With Multiple Tumors**

**Proband No.**	**Age (in Years) at Diagnosis of First Tumor (Second Tumor)**	**Phenotype**	**Metastatic Disease**	**Germline Genetic Analysis**	**Histology Reviewed**
1	63 (63)	Unilateral renal oncocytoma	No	No detectable mutation in *SDHA,SDHB*/*SDHC*/*SDHD,SDHAF2, MAX*, *TMEM127, FH, VHL*	No
		Unilateral PC			
2	76 (76)	Unilateral RCC	No	No detectable mutation in *SDHA, SDHB/SDHC/SDHD,SDHAF2, MAX, TMEM127, FH, VHL*	Yes
		Unilateral PC			
3	56 (56)	Unilateral RCC	No	No detectable mutation in *SDHA*, *SDHB*/*SDHC*/*SDHD,SDHAF2, MAX*, *TMEM127, FH, VHL*	Yes
		Unilateral PC			
		Breast carcinoma			
4	62 (64)	Unilateral PC	Yes (RCC)	No detectable mutation in *SDHA*, *SDHB*/*SDHC*/*SDHD,SDHAF2, MAX*, TMEM127, *FH, VHL*	No
		Multifocal RCC			
5	68 (68)	Unilateral PC	No	No detectable mutation in *SDHB* or *VHL*	No
		Unilateral RCC			
6	41 (41)	Carotid body PGL	No	Variant of uncertain significance	No
		Unilateral RCC		*SDHD* (c.34G>A p.Gly12Ser)	
				Tested for SDHB/C/D and VHL	
7	60 (60)	Unilateral RCC	No	No detectable mutation in *SDHB* or *VHL*	No
		Unilateral PC			
8	10 (26)	Abdominal PGL	No	*SDHB* mutation	No
		Unilateral RCC		c.141G>A (p.TRP47*)	
				Tested for VHL and SDHB	
9	62 (63)	Unilateral PC	No	*SDHB* mutation	No
		Unilateral RCC		c.268C>T (p.Arg90*)	
				Tested for SDHB, VHL	
10	43 (43)	Unilateral RCC	No	*MAX* mutation	No
		Unilateral PC		c.97C>T (p. Arg33*) Tested for *SDHA, SDHB, SDHC, SDHD, SDHAF2, TMEM127, MAX,* and *FH*	
11	53 (62)	Unilateral PCC	No	*TMEM127* mutation	Yes
		Unilateral RCC		c.117_120delGTCT (tested for *SDHA, SDHB, SDHC, SDHD, SDHAF2, TMEM127, MAX,* and *FH*)	
12	34 (39)	Carotid body HNPGL	Yes	*SDHB* mutation	Yes
		Unilateral RCC	(RCC)	c.79C>T (P.Arg27*).	
				Tested for *SDHB*, *SDHC*, *SDHD*, and *VHL*	

**Table 2.** **Clinical and Molecular Features of RAPTAS Kindreds With PC/PGL/HNPGL and a Renal Tumor in Two FDRs**

Proband No.	Age in Years at Diagnosis	Phenotype of Proband	Genetic Mutation Identified in Proband	Relative Affected	Phenotype of Relative
13	56	Renal oncocytoma	*SDHB* splice site intron mutation IVS1 + 1 G>T	Daughter (13)	Unilateral PC
14	50	HNPGL*^a^*	No detectable mutation in *SDHB* or *VHL*	Father (58)	Unilateral RCC
15	77	Unilateral PC	No detectable mutation in *SDHA*, *SDHB*/*SDHC*/*SDHD,SDHAF2, MAX*, *TMEM127, FH, VHL*	Daughter (51)	Unilateral RCC
16	57	HNPGL	No detectable mutation in *SDHA*, *SDHB*/*SDHC*/*SDHD,SDHAF2, MAX*, *TMEM127, FH, VHL*	Brother (54)	Unilateral RCC
17	57	Abdominal PGL*^a^*	*SDHB* mutation c.166-170delCCTCA (p.Pro56Tyrfs5X)	Brother (57)	Unilateral RCC
18	67	Abdominal PGL	No detectable mutation in *SDHB* or *VHL*	Brother (52)	Unilateral RCC
19	19	Unilateral PC	No detectable mutation in *SDHA*, *SDHB*, *SDHC*, *SDHD,SDHAF2, MAX*, *TMEM127, FH, VHL*	Father (65)	Unilateral RCC
20	60	Unilateral RCC	*SDHB* mutation c.380T>G (p.Ile127Ser)	Brother (64)	Unilateral PC
21	60	Unilateral PC	No detectable mutation in *SDHB* or *VHL*	2 brothers (50,63)	Unilateral RCC
22	27	Unilateral PC	No detectable mutation in *SDHB* or *VHL*	Father (49)	Unilateral RCC

^a^ Metastatic disease.

#### Clinical features of group B: familial non-VHL RAPTAS cases

Ten kindreds were identified containing two or more FDRs with PC/PGL/HNPGL and a renal tumor. Information including phenotype, genotype, and demographic information was available on 10 probands (6 females, 4 males) referred for genetic testing and basic demographic/phenotype information was available on the 11 affected FDRs (2 females, 9 males) (Table 2). Mean age at presentation of the probands was 56.6 years (SD, 17.3; range, 27 to 77 years) and mean age at tumor diagnosis in 11 affected FDRs was 52.3 years (SD, 16.3; range, 13 to 65 years). In eight kindreds, the proband presented with a PC/PGL/HNPGL (three with a PC, three with HNPGL, and two with abdominal PGL) and in two cases had malignant PGLs (an HNPGL and an abdominal PGL) (Table 2). Two probands presented with RCC and a renal oncocytoma. Most probands in group B had one affected relative, but one proband had two affected relatives (two brothers, both with RCC).

#### Molecular genetics analysis of the non-VHL RAPTAS case series

Molecular genetic analysis was performed on all 22 probands from groups A and B. All cases were tested for germline mutations in *VHL,* and *SDHB* and 8/12 (67%) of probands from group A and 6/10 (60%) of probands from cohort B were also tested for mutations in *SDHA*, *SDHC, SDHD*, *SDHAF2*, *FH*, *MAX*, and *TMEM127*. A germline *SDHB* mutation (four truncating mutations and a splice site mutation) was detected in 6/22 (27.3%) probands (three from group A and three from group B). Family testing was possible in two of three group B kindreds; in both cases, the affected relative harbored the *SDHB* mutation detected in the proband.

One proband was diagnosed with a variant in *SDHD* (c.34G>A, p.Gly12Ser) that was not considered pathogenic and did not prompt family screening. One proband presenting with RCC and unilateral PC age 43 years had a truncating mutation in the *MAX* gene (Table 1). This NGS result was confirmed by Sanger sequencing. Another proband from group A was found to have a truncating mutation in *TMEM127* (Table 1).

No statistically significant correlation was identified for younger age at first tumor diagnosis, PGL, renal oncocytoma or malignant PGL, and the identification of a genetic mutation (*P* > 0.05 for all associations). The mean MTS (11) value in group A patients with a mutation was 3.6 compared with 1.8 in those without a mutation (*P* = 0.09).

### Histology review

**Figure 1.**
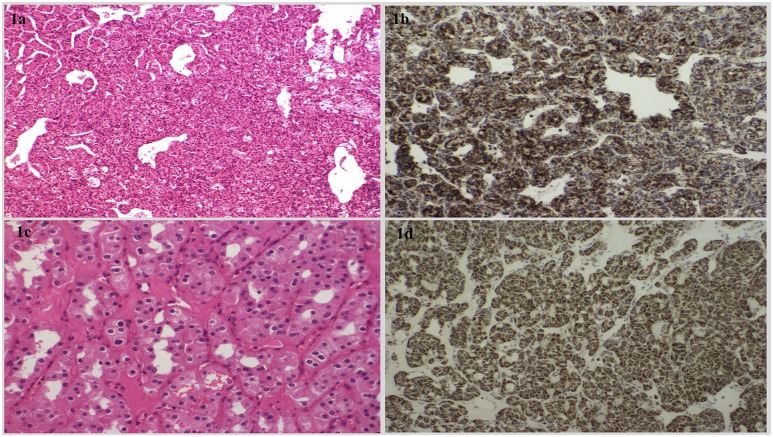
(a) The hematoxylin and eosin (H+E)-stained compact architecture and overall histological features consistent with a clear-cell RCC from proband 11 with a TMEM127 mutation. (b) Positive SDHB immunostaining in the same RCC tumor from proband 11. (c) Histological examination of a chromophobe RCC tumor from proband 3 with no detectable germline mutation (H+E staining ×200 high-power field). There is evidence of pleomorphic nuclei and perinuclear halos. (d) Positive SDHB immunostaining of the chromophobe RCC tumor.

**Figure 2.**
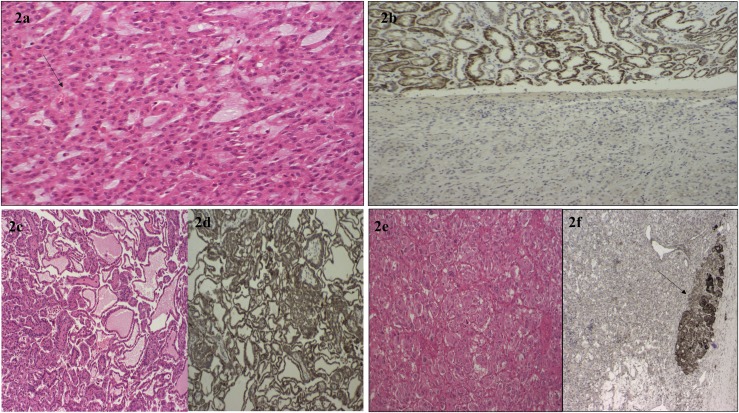
(a) The H+E-stained histological appearance of the SDHB-deficient RCC from proband 12. There is evidence of intracytoplasmic vacuoles marked by the black arrow. (b) Loss of SDHB protein expression on immunostaining of the RCC tumor from proband 12 in the lower part of the image, with SDHB staining present in the adjacent normal renal tissue visible in the upper image. (c) The histological appearances of a renal papillary carcinoma from proband 2 (H+E staining ×200 high-power field) and (d) preserved SDHB expression on immunostaining in this tumor. (e) A PC tumor from proband 2. (f) Negative SDHB immunostaining in the PC. The black arrow points to an area of normal adrenal tissue with preserved SDHB protein expression.

Archival tumor samples were available for four patients from group A (RCC samples from probands 2,3, 11, and 12 and a PC from proband 2) and histology review and SDHB immunostaining was performed (Figs. 1 and 2).

#### Tumor sequencing

Analysis of DNA extracted from the PC and RCC tumors from case 10 with the germline mutation in the *MAX* gene (c.97C>T p. Arg33*) revealed loss of heterozygosity (Supplemental Fig. 4), with higher reads in the mutant allele identified in the PC [reads wild-type/mutant: 77/151 (depth 228) and RCC (reads: 60/179, depth 239) compared with the germline (157/157, depth 314) [germline.v’s.PC *P* = 0.0002; germline.v’s.RCC *P* < 0.0001 (Fisher’s exact test)]. No additional somatic homozygous mutations were identified in other RAPTAS-related genes (*VHL*, *SDHA, SDHB*, *SDHC*, *SDHD*, *FH*, *TMEM127*) in either tumor from case 10 (Supplemental Table 4).

Loss of SDHB immunostaining in the PC from case 2 prompted additional sequencing of tumor tissue from the PC and RCC because germline testing did not reveal a germline mutation in *SDHx or VHL* (Table 1). No somatic mutation in *SDHA*/*SDHB*/*SDHC*/*SDHD* was identified in either tumor, but a somatic variant (not present in the germline) in *VHL* (c.245G>T p Arg82Leu) was identified in the PC tumor but not the RCC from case 2.

### Literature review

Germline mutations in at least 25 different genes have been reported to predispose to PC/PGL/HNPGL or RCC (*NF1, RET, MAX, EGLN1, EGLN2, MSH2, KIFIB, SDHAF2,MEN1, BAP1, CDC73, CDKN2B, FLCN, MET, PBRM1, PTEN, TSC1, TSC2, FH, SDHA, SDHB, SDHC, SDHD, TMEM127*, and *VHL*) (6–9, 12–14, 15). However, with the exception of VHL disease and, to a lesser extent, *SDHB* mutations (16–20), other reported genetic causes of RAPTAS (as defined here) are rare.

A total of 21 kindreds and 39 patients were identified from the literature with a non-VHL RAPTAS phenotype and a germline mutation. Mean age of tumor onset was 36.1 years (17 to 61 years) [31.8 years (17 to 47 years) for PC/PGL/HNPGL and 41.4 (19 to 61 years) years for RCC]. The most commonly mutated gene was *SDHB* (16/21 kindreds) and 44% (7/16) had a deletion (missense in 5/16 and truncating mutations in 4/16). Most reported cases were diagnosed with an RCC. Metastatic RCC was reported in five patients with *SDHB* mutations, one patient with an *SDHC* mutation, and one patient with an *SDHD* mutation. Three cases had bilateral RCC and one bilateral PC. Metastatic PC/PGL/HNPGL occurred in one patient. Renal oncocytoma was described as part of three cases of RAPTAS (two with an *SDHB* mutation and one with a *MAX* mutation) (Table 3).

**Table 3.** **Clinical and Molecular Genetic Features of Non-VHL RAPTAS Cases Identified in the Literature**

Gene	Mutation	Group A/B	(PC/PGL/HNPGL) Location (Age)	RCC Tumor Type (Age in Years)	Sex	Tumor of Relative (Age in Years)	Reference
*SDHB*	c.3G>A (p.Met1Ille)	A+B	PGL (25)	Bilateral RCC (25)	M	RCC, brother (23)	13
*SDHB*	c.3G>A (p.Met1Ille)	B	No	Unilateral RCC (23)	M	RCC, PGL, brother (25)	13
*SDHB*	Exon 3 deletion	A	HNPGL (30)	Unilateral RCC (36)	M		13, 33
*SDHB*	c.166-170 del CCTCA (p.Pro56TryfsX5)	A	PGL (28)	Unilateral RCC (28)	M		33
*SDHB*	C.423+1G>A	B	No	Unilateral RCC		PC, brother (44)	7, 33
*SDHB*	Exon 1 deletion	B	No	Unilateral RCC*^a^* (36)	M	RCC, brother (25)*^a^*	39
*SDHB*	Exon 1 deletion	A+B	PC	Unilateral RCC (42)	F	PGL, sister	39
*SDHB*	268C>T (p.Arg90X)	A+B	PGL	Unilateral RCC (61)	M	PGL, son	33
*SDHB*	c.286G>A (p.Gly96Ser)	B	No	Unilateral RCC (52)*^a^*	F	RCC, daughter	39
*SDHB*	c.541-2A>G	B	No	Unilateral RCC (19)	F	PGL, mother	39
*SDHB*	c.689G>A (p.Arg230His)	B	No	Unilateral RCC (52)	F	PGL, daughter	39
*SDHB*	c.541-2A>G	B	No	Unilateral RCC (50)	M	RCC, brother*^a^*	39
*SDHB*	Del exon 1	A	PGL (17)	Unilateral renal oncocytoma	F		39
*SDHB*	c.170A>G (p.His57Arg)	B		Unilateral RCC*^a^* (28)	M	PGL, mother*^a^*	20
*SDHB*	c.847-50delTCTC	A+B	Unilateral RCC (26)	PGL	M	RCC, PGL, brother (24)	20
*SDHC*	c.397C>T (p.Arg133X)	B	No	Unilateral RCC (53)*^a^*	F	RCC, son (40)	39
*SDHC*	c.3G>A (p.Met1I)	B	HNPGL (46)	Bilateral RCC (48,60)	M	Bilateral RCC, mother (48,60)	40
*SDHD*	c.239G>T (p.Leu80Arg)	A+B	Bilateral HNPGL (17), PGL(28)	Unilateral RCC (45)*^a^*	M	HNPGL, father, PC brother	39
*TMEM127*	c.308delG (p.Gly103Alafs)	A	PC (47)	Unilateral RCC (47)	F		6
*MAX*	Deletion exon 1+2	A+B	Bilateral PC (45)	Unilateral oncocytoma (45)	M	Bilateral PC, brother (28)	8

Abbreviations: F, female; M, male.

^a^ Metastatic disease.

In addition to patients with RAPTAS, separate case reports of PC/PGL/HNPGL or renal tumors have been reported in association with the six genes described in Table 3, as well as with mutations in *FH* (14, 21) and *SDHA* (9, 22) (although no cases of coexisting PC/PGL and RCC in the same patient had been reported in conjunction with a mutation in *FH/SDHA*). Although there are very rare cases of tuberoses sclerosis and neurofibromatosis type 1 with a PC or RCC, respectively, these do not cause diagnostic difficulties because of the syndromic features in such cases and have not been reported to cause RAPTAS (23, 24).

## Discussion

A large case series and literature review demonstrated that non-VHL RAPTAS is genetically heterogeneous. RAPTAS may be caused by germline mutations in six genes (*VHL, SDHB, SDHC, SDHD, TMEM127*, and *MAX*) and two further genes, *FH* and *SDHA*, have each been reported to predispose to both groups of tumors (PC/PGL/HNPGL and renal tumors) and may yet be described as a cause of RAPTAS. Also, germline mutations in *MET* cause familial type 1 papillary RCC and recently *MET* variants have been linked to PC/PGL susceptibility (5).

In both the literature review and case series, *SDHB* mutations were the most common identified cause of non-VHL RAPTAS. Less frequently, RAPTAS was associated with mutations in other SDHx genes and mutations in *TMEM127* and *MAX*. A limitation of this case series was that all cases had not been tested for mutations in the rarer RAPTAS genes (*SDHC*, *TMEM127*, and *MAX*) and a limitation of the literature review is probable bias against reports of RAPTAS without an identified genetic diagnosis. Nevertheless, we found that there is a substantial group of RAPTAS patients without an identified germline mutation, suggesting that further RAPTAS genes are still to be identified.

Recently Kopershoek *et al.* (8) described a germline *MAX* mutation (a large, complex genomic alteration encompassing the intragenic and promoter regions of *MAX* and *FUT8*) in a patient with renal oncocytoma, bilateral PC, and erythrocytosis and two siblings with bilateral PC. In this study, we report the association of RCC with a germline *MAX* mutation (c.97C>T p. Arg33*). We detected evidence of preferential loss of the wild-type allele in both tumors (PC and RCC) similar to previously reported cases of *MAX*-related tumors PC/PGL (25). This finding expands the phenotype associated with *MAX* mutations and raises the intriguing possibility that *MAX* may be a candidate gene for inherited RCC [*SDHB* mutations were originally described in association with PC/PGL/HNPGL (26), then with RAPTAS (20), and then familial RCC-only (16) phenotypes]. Although mutations in all RAPTAS genes are inherited in autosomal dominant manner, mutations in *MAX* and *SDHD* show a parent-of-origin–dependent tumorigenesis, and tumors occur almost exclusively following paternal transmission of the mutation. Hence the clinical management and genetic counseling of RAPTAS kindreds with *SDHD* and *MAX* mutations will differ from those with mutations in other RAPTAS genes.

We describe the second reported case of a patient with RAPTAS resulting from a mutation in *TMEM127*. The first report was in a 47-year-old woman (6) with multifocal unilateral PC and a unilateral (clear cell) RCC (6). A germline deletion mutation in *TMEM127* (c.308delG) and an additional germline variant in *SDHB* (159_*184delins25) was identified in this patient, but SDHB immunohistochemistry showed preservation of SDHB expression in both tumors. Histology of the RCC in RAPTAS patient 11 with a *TMEM127* mutation demonstrated a clear-cell RCC. Although this is the most common type of RCC, the four additional reported cases of *TMEM127*-associated RCC were all clear-cell variant RCC (27).

### Role of clinical features in suggesting specific genes

In genetically heterogeneous conditions, it is helpful if specific clinical features can guide genetic testing. Clear-cell RCC, PC (less often PGL and rarely HNPGL), and retinal and central nervous system hemangioblastomas (15) (or the presence of pancreatic or renal cysts) should prompt genetic testing for *VHL* mutations. The occurrence of HNPGL, abdominal PGL, and malignant PPGL or the co-occurrence of wild-type gastrointestinal stromal tumors suggests a possible SDHx mutation. Adrenal PC is more common in VHL disease, whereas (extra-adrenal) PGL with *SDHB* disease but with a secretory pattern (predominantly noradrenergic) is similar to *VHL* and *SDHX* and there are similar features on positron emission tomography computed tomography with tracers such as 18-fluorodeoxyglucose (28).

Indicators of an inherited cancer predisposition syndrome include the occurrence of uncommon/rare tumors in the same individual, related tumor types in close relatives, early age at diagnosis, and the presence of multicentric disease. In patients with RCC, genetic investigation should be considered in sporadic cases age ≤45 years (29). Although the literature review identified patients with non-VHL RAPTAS and a germline mutation had relatively young-onset PC/PGL/HNPGL (mean, 31.8. years; RCC, 41.4 years) in the case series, there was no clear relationship between age at tumor diagnosis and presence/absence of a mutation. Although the difference in MTS (11) between mutation-positive and mutation-negative cases did not reach statistical significance, further studies are required to determine MTS utility in group A RAPTAS cases. Although RAPTAS might in some cases arise coincidentally, we note in two *SDHB* mutation-positive cases in our series (probands 9 and 18), age at tumor diagnosis was 60 years or older. Therefore, we would suggest that either all cases of RAPTAS should undergo molecular investigation or the cutoff for age at tumor diagnosis for not pursuing genetic testing should not be <70 years.

### Role of histology in suggesting specific genes in RAPTAS

**Figure 3.**
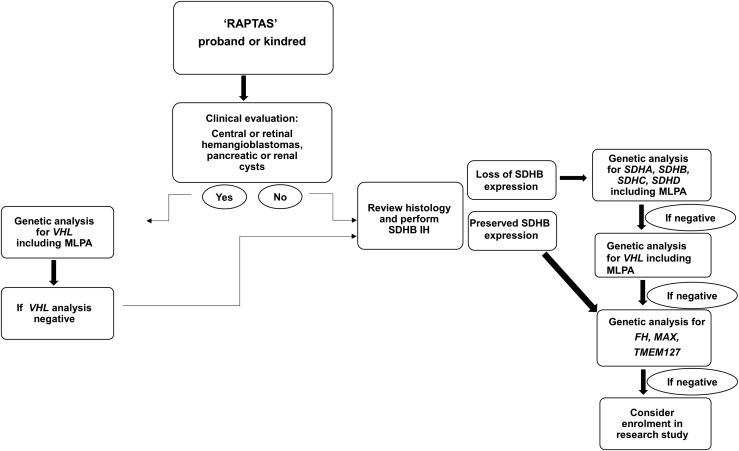
Flowchart of the recommended genetic evaluation of potential RAPTAS kindreds. MLPA, multiple ligation-dependant probe amplification.

Histopathological features may be used to prioritize likely genetic causes of RAPTAS (15). For example, VHL mutations are almost invariably associated with clear-cell RCC (30), and a unique morphology consisting of solid architecture, distinctive intracytoplasmic inclusions, and intratumor mast cells is characteristic of SDHB-deficient RCC (31, 32) (Fig. 2a). Immunohistochemistry is a useful diagnostic adjunct because SDHB-deficient RCC shows negative immunoreactivity (33) (Fig. 2b). Interestingly, proband 2 had evidence of succinate dehydrogenase deficiency on SDHB immunostaining of the PC (Fig. 2c), but immunostaining showed preserved SDHB expression in the RCC tumor. Sequencing of both the PC and RCC tumors in case 2 revealed a somatic mutation in *VHL* (c.245G>T p Arg82Leu) in the PC but not the RCC, with no evidence of mutation in *SDHA/SDHB/SDHC/SDHD* genes. False-positive results using SDHB immunohistochemistry (as apparently occurred in this case) have been reported for patients with germline VHL mutations (34) (Fig. 2d). A potential alternative explanation for the discrepant SDHB immunohistochemistry results in case 2 is that the first hit is an undetected germline *VHL* mutation (*e.g.*, intronic mutation, copy number alteration) and that the somatic *VHL* missense mutation in the PCC was the “second hit.” However, the RCC histology was a papillary (Fig. 2c), whereas renal tumors in VHL disease are clear cell (18, 35). Nevertheless, it is important to consider that VHL mutations can lead to false-positive results on SDHB immunohistochemistry (34); therefore, we recommend that those patients with RAPTAS, without a detectable germline mutation in *SDHx*, but with loss of SDHB immunoexpression on tumor studies, undergo genetic screening for *VHL* mutations (Fig. 3).

### Molecular pathways implicated in different genetic causes of RAPTAS

Transcriptome profiling separates inherited PC/PGL into two categories (5). First, the “pseudohypoxic cluster” with the upregulation of hypoxia signaling pathways and “cluster 2” is characterized by an upregulation of kinase signaling pathways (5). *VHL*, *FH*, or *SDHx-*associated PC/PGL fall into the pseudohypoxic cluster 1 (5). SDHx and FH inactivation leads to the accumulation of oncometabolites such as succinate and fumarate that inhibit alpha ketoglutarate dependant dioxygenase enzymes, promoting stabilization of hypoxia inducible factor complex (5) and inhibiting histone and DNA demethylation enzymes, resulting in DNA hypermethylation (36).

Cluster two gene mutations (*RET*, *NF1*, *TMEM127*, *MAX*) activate the MAPK and phosphatidylinositol 3-kinase–AKT–mTOR pathways (5). *MAX* is a protein that contains a basic helix loop helix zipper commonly involved in a complex formation and sequences in the promoter region of hundreds of genes encoding for proteins essential in cellular metabolism and angiogenesis (37).

### Investigation of potential RAPTAS patients

In study group A patients, one-third presented with PC/PGL/HNPGL and were subsequently diagnosed with an RCC. The longest interval between the presenting tumor (abdominal PGL) and diagnosis of RCC was 16 years (proband 8). For the other three patients, mean interval between the first tumor and RCC was 4 years (median, 2 years; range, 1 to 9 years). Recently published European guidelines recommend a 10-year follow-up for patients with sporadic PC and life-long follow-up for patients with PGL/HNPGL or those patients with a confirmed genetic predisposition. The recommendations for follow-up include biochemical and radiological surveillance that would include abdominal imaging capable of detecting renal tumors. Data from this study suggest that this surveillance protocol will facilitate the detection of patients with RAPTAS (38).

Patients meeting our clinical criteria for RAPTAS should be referred for genetic testing. If gene panel testing is not available/undertaken, then single-gene testing should be prioritized as suggested in Fig. 3. It is important that *SDHB* mutation analysis includes investigation for exonic deletions/duplications because the literature review revealed a higher than expected proportion of deletions in *SDHB*-associated RAPTAS. Mutation-positive cases should receive appropriate follow-up and surveillance. The clinical benefits of identifying germline genetic variants in patients with non-VHL RAPTAS also include family screening, and, in the future, genetic classification may facilitate a personalized treatment approach.

## Conclusion

We described the largest cohort to date of non-VHL RAPTAS and have undertaken a literature review of reported cases. The term RAPTAS (rather than inherited PC-PC) emphasizes that many cases may be sporadic with no family history and that not all cases may have a genetic origin. We provide guidelines for genetic testing in suspected RAPTAS and for clinical diagnostic criteria that include both sympathetic and parasympathetic PGL (with PC) in the criteria and both malignant (RCC) and benign (oncocytoma) renal tumors. Application of whole-exome and whole-genome sequencing to undiagnosed RAPTAS cases will provide further insights into the molecular mechanisms of this association and improve the management of these cases.
