# Supplementary material for: Clinical and Molecular Features of Renal and Pheochromocytoma/Paraganglioma Tumor Association Syndrome (RAPTAS): Case Series and Literature Review
Source: J Clin Endocrinol Metab. 2017 Jul 28;102(11):4013–22. doi: 10.1210/jc.2017-00562 (PMC5673270; doi:10.1210/jc.2017-00562)

Supplementary data

Methods:

- **Histology review:**

Central renal tumour pathology review was undertaken (when suitable samples were available) by an experienced uro-pathologist (AW). The purpose of histology review was to identify suspicious pathological features, which may be associated with, or predict, non-VHL RAPTAS. The pathologist was blinded to the mutational status and SDHB immunohistochemistry was performed on 4um sections of paraffin embedded tissue using a commercially available (Sigma Aldrich, United Kingdom) SDHB polyclonal rabbit antibody at a dilution of 1:300.

- **Bioinformatics analysis**

All samples were aligned to the hg38 version of the reference genome using bwa 0.7.15. The generated SAM file was compressed into a BAM file and sorted by genomic position using samtools 1.3.1. The mean depth of read for all samples across all sequenced genes was 1860.1. In germline/tumour pairs from the same individual the variant calling was performed using GATK 3.7 MuTect2 Algorithm. All variants with a Q prhed scaled quality score lower than 30 or a depth of read lower than 30 were excluded from further analysis. The generated VCF files were manipulated using VCFTools 0.1.13 and annotated using annovar.

Table S4: The Ion AmpliSeq™ Cancer Panel targets 68 genes

| MPL | NRAS | ATP1A1* |
| --- | --- | --- |
| SDHC* | EGLN1* | FH* |
| ALK | EPAS1* | TMEM127* |
| IDH1 | ERBB4 | VHL |
| MLH1 | CTNNB1 | CACNA1D* |
| PIK3CA | FGFR3 | PDGFRA |
| KIT | KDR | FBXW7 |
| SDHA* | APC | CSF1R |
| NPM1 | EGFR | MET |
| SMO | BRAF | EZH2 |
| FGFR1 | JAK2 | CDKN2A |
| GNAQ | ABL1 | NOTCH1 |
| RET | PTEN | FGFR2 |
| HRAS | SDHAF2* | MEN1* |
| ATM | SDHD* | KCNJ5* |
| KRAS | PTPN11 | HNF1A |
| FLT3 | RB1 | MAX* |
| AKT1 | IDH2 | CDH1 |
| TP53 | NF1* | ERBB2 |
| SMAD4 | STK11 | GNA11 |
| PRKACA* | JAK3 | SMARCB1 |
| SRC | GNAS | SDHB* |
| ATP2B3* | KIF1B* |  |

*= additional custom hot spot gene regions added.

- **Figure S4:** This figure illustrates preferential loss of the wild type allele in both phaeochromocytoma (PCC) and RCC tumours from patient #10 with the identified germline mutation in *MAX* (c.97C>T p. Arg33*). This figure shows the read count of the wild type/mutant allele (G/A) demonstrated in the BAM files viewed on integrative genomics viewer (IGV) from the i) germline (157/157, depth 314), ii) PCC (reads wild type/mutant: 77/151, depth 228), iii) and RCC (reads: 60/179, depth 239) tumours from patient #10.


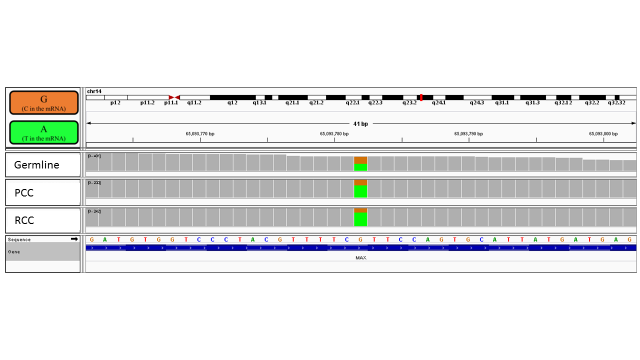


- **Figure S5**: Evidence of a somatic VHL mutation (c.245G>T p Arg82Leu) in the PCC tumour from case two with no evidence of this mutation in germline or RCC tumour in case 2 on analysis of BAM files on IGV.


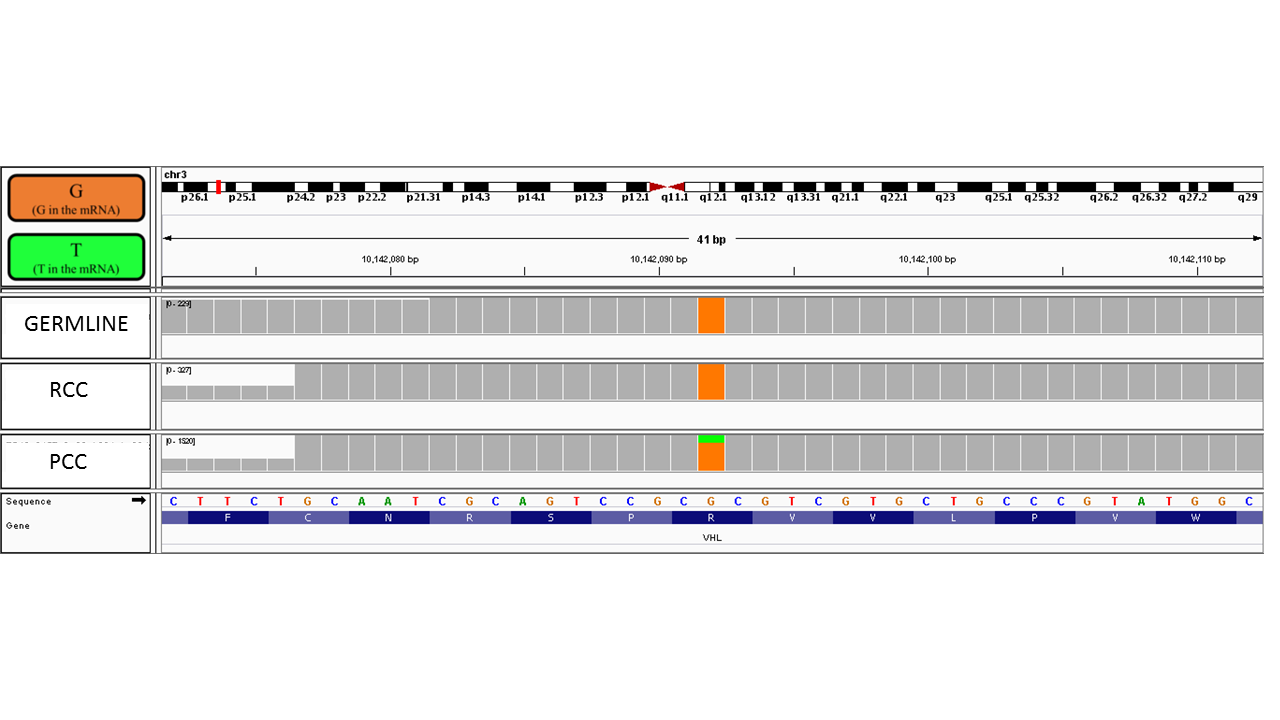

Supplement: Supplementary file 1 [file jc.2017-00562.sd1.docx]
